# Automatic Image Selection Model Based on Machine Learning for Endobronchial Ultrasound Strain Elastography Videos

**DOI:** 10.3389/fonc.2021.673775

**Published:** 2021-05-31

**Authors:** Xinxin Zhi, Jin Li, Junxiang Chen, Lei Wang, Fangfang Xie, Wenrui Dai, Jiayuan Sun, Hongkai Xiong

**Affiliations:** ^1^ Department of Respiratory Endoscopy, Shanghai Chest Hospital, Shanghai Jiao Tong University, Shanghai, China; ^2^ Department of Respiratory and Critical Care Medicine, Shanghai Chest Hospital, Shanghai Jiao Tong University, Shanghai, China; ^3^ Shanghai Engineering Research Center of Respiratory Endoscopy, Shanghai, China; ^4^ School of Electronic Information & Electrical Engineering, Shanghai Jiao Tong University, Shanghai, China; ^5^ Department of Ultrasound, Shanghai Chest Hospital, Shanghai Jiao Tong University, Shanghai, China

**Keywords:** endobronchial ultrasound, strain elastography, machine learning, lymph nodes, image selection

## Abstract

**Background:**

Endoscopic ultrasound (EBUS) strain elastography can diagnose intrathoracic benign and malignant lymph nodes (LNs) by reflecting the relative stiffness of tissues. Due to strong subjectivity, it is difficult to give full play to the diagnostic efficiency of strain elastography. This study aims to use machine learning to automatically select high-quality and stable representative images from EBUS strain elastography videos.

**Methods:**

LNs with qualified strain elastography videos from June 2019 to November 2019 were enrolled in the training and validation sets randomly at a quantity ratio of 3:1 to train an automatic image selection model using machine learning algorithm. The strain elastography videos in December 2019 were used as the test set, from which three representative images were selected for each LN by the model. Meanwhile, three experts and three trainees selected one representative image severally for each LN on the test set. Qualitative grading score and four quantitative methods were used to evaluate images above to assess the performance of the automatic image selection model.

**Results:**

A total of 415 LNs were included in the training and validation sets and 91 LNs in the test set. Result of the qualitative grading score showed that there was no statistical difference between the three images selected by the machine learning model. Coefficient of variation (CV) values of the four quantitative methods in the machine learning group were all lower than the corresponding CV values in the expert and trainee groups, which demonstrated great stability of the machine learning model. Diagnostic performance analysis on the four quantitative methods showed that the diagnostic accuracies were range from 70.33% to 73.63% in the trainee group, 78.02% to 83.52% in the machine learning group, and 80.22% to 82.42% in the expert group. Moreover, there were no statistical differences in corresponding mean values of the four quantitative methods between the machine learning and expert groups (p >0.05).

**Conclusion:**

The automatic image selection model established in this study can help select stable and high-quality representative images from EBUS strain elastography videos, which has great potential in the diagnosis of intrathoracic LNs.

## Introduction

The differential diagnosis of malignant and benign intrathoracic lymph nodes (LNs) is an important medical problem related to the diagnosis and prognosis of intrathoracic diseases. Compared with surgical examination, needle techniques are recommended as the first choice to obtain tissues (1, 2). Endobronchial ultrasound guided transbronchial needle aspiration (EBUS-TBNA) is an important minimally invasive tool to evaluate the benign and malignant intrathoracic LNs.

Previous literature mentioned that ultrasonographic features were suggested to be used for predicting benign and malignant diagnosis of patients undergoing EBUS-TBNA (3). EBUS imaging includes three modes of grayscale, blood flow Doppler and strain elastography. Studies indicated that strain elastography had the best diagnostic value among the three modes (4, 5). Elastography has been widely used in breast lesions, thyroid, pancreas, prostate, liver and endoscopic ultrasound (6–11). Through exerting repeated and slight pressure on the examined lesions, elastography can quantify the elasticity of tissues by measuring the deformation and present it in the form of various colors (12–14). The colors from yellow/red, green to blue represent tissues from lower to higher relative stiffness, respectively (13). Malignant infiltration of tumor cells can alter cell morphology and overall histology of tissues resulting in a stiffer property. Elastography can indirectly predict malignant lesions by reflecting its relative stiffness (15). EBUS strain elastography plays an important role in differentiating intrathoracic benign and malignant LNs (16). The bronchoscopist can select the target LN and possible metastatic sites within the LN for biopsy according to strain elastography during EBUS-TBNA (17, 18).

With respect to qualitative analysis of strain elastography image, the five-score grading method had specific classification and when score 1–3 was defined as benign and score 4–5 as malignant, the diagnostic accuracy in predicting malignant LNs can reach 83.32% (4). Quantitative methods include stiff area ratio (SAR), elasticity ratio of blue/green (B/G), mean hue value and mean gray value can comprehensively evaluate the quality of elastography images (4, 5, 18–23). Qualitative methods are more convenient for clinical application, but strong subjectivity exists inevitably. Therefore, doctors with various experience may have different judgement on the same strain elastography video. Although the quantitative method are relatively objective, the images used for quantitative analysis are still selected subjectively. Moreover, ultrasound imaging is the specialty of ultrasound doctors, and endoscopists may not make full use of strain elastography due to the limited experience.

In recent years, with the development of machine learning algorithm, machine learning has shown an important role in the field of medical imaging with favorable performance, such as skin cancer, retinal fundus photographs, gastrointestinal endoscopy, chest CT and other aspects (24–27). By extracting multiple quantitative image features which may be difficult for doctors to observe, machine learning can give a likelihood of each case and classify images accurately. Research demonstrated that machine learning combined with colorectal endoscopy for colorectal lesions diagnosis was comparable to that of experts (28). In the field of bronchoscopy, a computer-assisted diagnosis (CAD) system has been used to classify normal mucosa, chronic bronchitis and lung tumors under the white-light bronchoscopy, which achieved a classification rate of 80% (29). In addition, a machine learning texture model can get an accuracy of 86% in classifying cancer subtypes using bronchoscopic findings (30). However, there are few applications of machine learning on EBUS strain elastography. Therefore, the purpose of this study was to establish a machine learning model which can realize automatic selection of representative images from strain elastography videos.

## Materials and Methods

### Patients and LNs

Patients who met the following criteria and underwent EBUS-TBNA examination in Shanghai Chest Hospital from June to December 2019 were enrolled in this study (1): At least one enlarged intrathoracic LNs (short axis >1 cm) based on computed tomography (CT), or at least one positive ^18^F-FDG uptake detected (standardized uptake value >2.5) by positron emission tomography (2); Pathological confirmation was clinically required and EBUS-TBNA examination was feasible (3); Patients who did not have contraindications to EBUS-TBNA and signed informed consent. LNs with qualified strain elastography videos were analyzed in the study. LNs in December were used as the test set to assess the automatic representative images selection model and the remained were used as the training set and validation set. This study was approved by the local Ethics Committee of Shanghai Chest Hospital (No. KS1947) and registered at ClinicalTrials.gov PRS (NCT04328792).

### EBUS Strain Elastography Procedure

LNs were examined by the ultrasound bronchoscopy (BF-UC260FW, Olympus, Tokyo, Japan) and EBUS strain elastography videos were recorded by the ultrasound processor (EU-ME2, Olympus, Tokyo, Japan). The operator detected the location of the target LN and measured the EBUS size at the maximal cross-section of grayscale mode. After observing the grayscale and blood flow Doppler modes, the operator switches to the strain elastography mode and elastography imaging was formed through the patient’s respiration, cardiac impulse and blood vessel pulse generally. In the case of unsatisfactory imaging, the operator shall exert appropriate pressure to the target LN by pressing the up-down angle lever of bronchoscope at a frequency of three to five times per second to obtain better imaging. The maximal cross-section of the LN was recorded and two 20-second videos were saved (4). Subsequently, EBUS-TBNA was performed to obtain the cytological specimens for pathological examination. All operators retained strain elastography videos and sampled LNs according to the above standard steps. The final diagnosis of LNs was determined on EBUS-TBNA, thoracoscopy, mediastinoscopy, transthoracic thoracotomy or other pathological examinations, microbiological examination or clinical follow-up for more than one year.

### Development of Automatic Representative Images Selection Model for Strain Elastography Videos

The training set and validation set were randomly divided at a quantity ratio of 3:1 to train the model with optimal hyper-parameters. The same proportion of benign and malignant LNs was maintained in the two datasets. We developed models with various values of hyper-parameters on the training set and assessed these models on the validation set to determine the hyper-parameter according to the performance. Once the hyper-parameter was determined, we used both the training set and validation set to train the model for prediction and evaluation on the test set. In this paper, the hyper-parameters included the number of representation patterns and whether adopting the update-and-predict strategy or not. The candidate numbers of representation patterns included 32, 64, and 128. Blind to the final diagnosis of LNs, two experts with experience of EBUS images observation >500 LNs assessed the image quality of the validation sets together as following: score 1 (scattered soft, mixed green-yellow-red), score 2 (homogeneous soft, predominantly green), score 3 (intermediate, mixed blue-green-yellow-red), score 4 (scattered hard, mixed blue-green), score 5 (homogeneous hard, predominantly blue). Scores 1–3 are classified as benign and 4–5 as malignant (4). Four quantitative methods were also used to verify the diagnostic performance of the validation sets. Assessments on the validation set showed that we could yield the highest accuracy when adopting run-twice strategy and using 64 representative patterns (**Supplementary Tables 1** and **2**). When we trained the model with determined hyper-parameters, we used it to make prediction on the test set. Note that the test set is not used in the phase of training.

**Figure 1:**
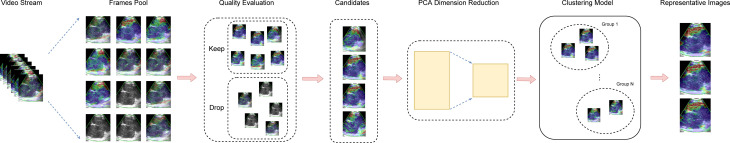
The process of automatic selection of representative images. Frames were extracted from the video stream to construct a frame pool initially. Then, inferior frames are dropped during the quality evaluation procedure, and the eligible frames are kept as candidates for representative images. Next, the PCA was employed for dimension reduction. Ultimately, the clustering model select representative images from candidates. PCA, principal component analysis.

**Figure 1** illustrated the process of representative strain elastography images selection with the proposed machine learning algorithm. Initially, the elastography video was converted into a sequence of frames with quality evaluated. According to the proportion of colored pixels and relative intensity of a frame (**Supplementary Material** and **Supplementary Figure 1**), the original frames were divided into qualified and unqualified, and only qualified frames were kept for succeeding procedures. Additionally, to avoid overwhelming qualified frames and reduce complexity, the adjacent two frames of selected qualified frame were dropped. Then, feature engineering was performed on the remaining frames. We constructed the features of each frame with the 512 bin color histogram to describe the color distribution of elastography images (31). Further, the principal component analysis (PCA) algorithm was applied to reduce the feature dimension, and a 40-dimension feature space was obtained. The number of dimensions depended on the training set, and 40-dimension was capable to keep 99% component in this study. Clustering plays an important role in video analysis (32–35). Considering the selective principle of experts that the most repeatable pattern across the video is selected as representative frames, we employed the k-means clustering algorithm in this study. In the phase of training, the k-means clustering was performed on the training set to obtain representative patterns (cluster centers). In the phase of prediction, the frame features from the test video were allocated to patterns extracted from the training set. Given a test video, the pattern owning most frames was regarded as the representative pattern and three frames closest to the representative pattern were selected as the representative images.

In real-world applications, however, it is hard to collect a training set that has sufficient examples to cover all possible situations and guarantee the generalization ability of the trained model. Consequently, a limited training set usually leads to a performance gap, when applied to the real data. To narrow this gap, in the phase of prediction, we proposed an update-and-predict strategy that ran the trained model twice on the test set. The first run produced the initial predictions of test videos which were used for updating the cluster centers in the model. Subsequently, the updated model was used to obtain the final predictions on the test set. Note that the K-means clustering is an unsupervised learning algorithm that does not require manual annotation or ground truth. Therefore, we leveraged K-means clustering in this paper to update our model using only the predictions of test videos rather than accessing their labels. The label information was not leaked in the phase of prediction. As a result, we can narrow the gap between the training set and test set and do not cause the leakage of label (supervision) information by using the update-and-predict strategy.

### Evaluation of Representative Images

**Figure 2:**
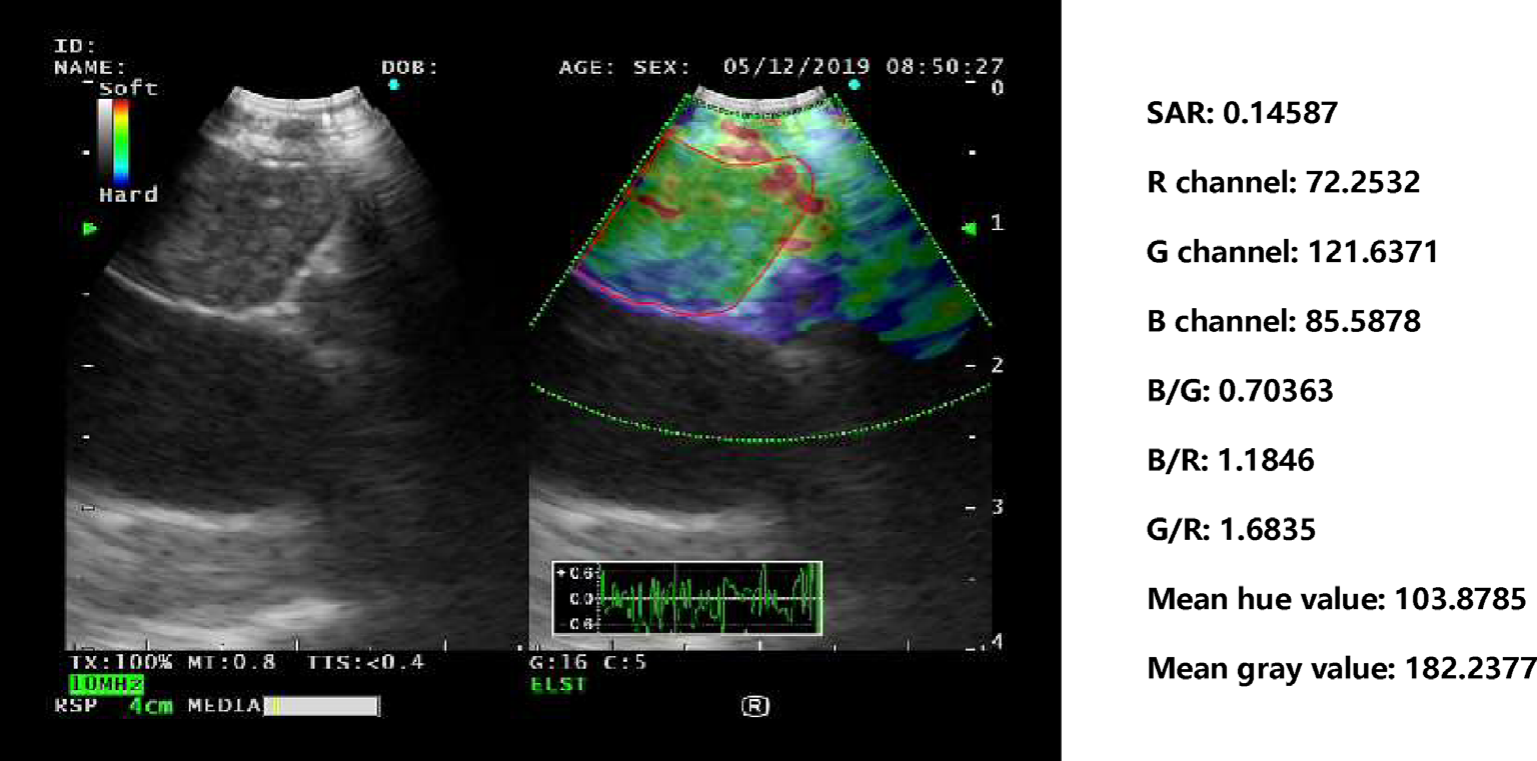
Delineation of ROI on strain elastography images and output of quantitative parameters. Representative images of the machine learning group, expert group and trainee group were all input into a computer program developed by Matlab™. The schematic diagram showed an elastography image of a LN with nonspecific lymphadenitis in 4R station, and ROI was outlined on the elastography image. Results of the four quantitative methods including SAR, RGB, mean hue value and mean gray value were then output by the program. ROI, region of interest; LN, lymph node; SAR, stiff area ratio; B/G, elasticity ratio of blue/green; B/R, elasticity ratio of blue/red; G/R, elasticity ratio of green/red.

For the three images selected by the automatic image selection model on the test set, the same two experts evaluated grading score together. Expert group and trainee group (experience of CP-EBUS image observation less than 30 LNs) were employed to select representative images which were used for comparison with that of machine learning. The three experts reviewed two elastography videos of each LN and selected one representative image for qualitative evaluation, respectively. Qualified images shall cover the maximal cross-section of the target LN and have good repeatability (4). Three trainees selected representative frames and evaluated qualitative score of corresponding pictures in the same way. The quantitative measurement of the three groups of images was operated by the elastography quantitative system (Registration number: 2015SR191866) developed by Matlab and the region of interest was outlined by an expert (**Figure 2**). Results of four quantitative methods including SAR, B/G, mean hue value and mean gray value were output by the program. The first method SAR was the ratio of blue pixels to pixels of the whole LN (5, 18–20). RGB is a color space model which represents the red, green and blue channel colors, and B/G was calculated in this study (21). Hue histogram analysis was performed for selected images and the third method mean hue value corresponds to the global elasticity of the LN (22). The fourth method mean gray value has been studied in the diagnosis of breast cancer and intrathoracic LNs (4, 23). All above procedures carried by experts and trainees were in the situation of blind to the clinical information and pathological results of target LNs.

### Statistical Analysis

For qualitative score, the Friedman test was used for the differences among the three images selected by the automatic image selection model and experts, and the Wilcoxon signed-rank test was used for the pair comparison. For quantitative variables, receiver operating characteristic (ROC) curve was used to obtain the area under the curve (AUC) and the cut-off value with the best diagnostic performance. The paired t-test was used for quantitative mean values comparison between images of the machine learning model and experts. The stability of the quantitative results within the three groups was evaluated using the coefficient of variation (CV), and the comparison of the CV among the three groups was further analyzed by the paired t-test. The p value <0.05 was considered statistically significant for above statistical analyses. Sensitivity, specificity, positive predictive value (PPV), negative predictive value (NPV), and accuracy for differentiating benign and malignant LNs were calculated by the corresponding formulas. All statistical analyses were performed by SPSS version 25.0 (IBM Corp., Armonk, NY, USA).

## Results

### Patients and LNs

**Figure 3:**
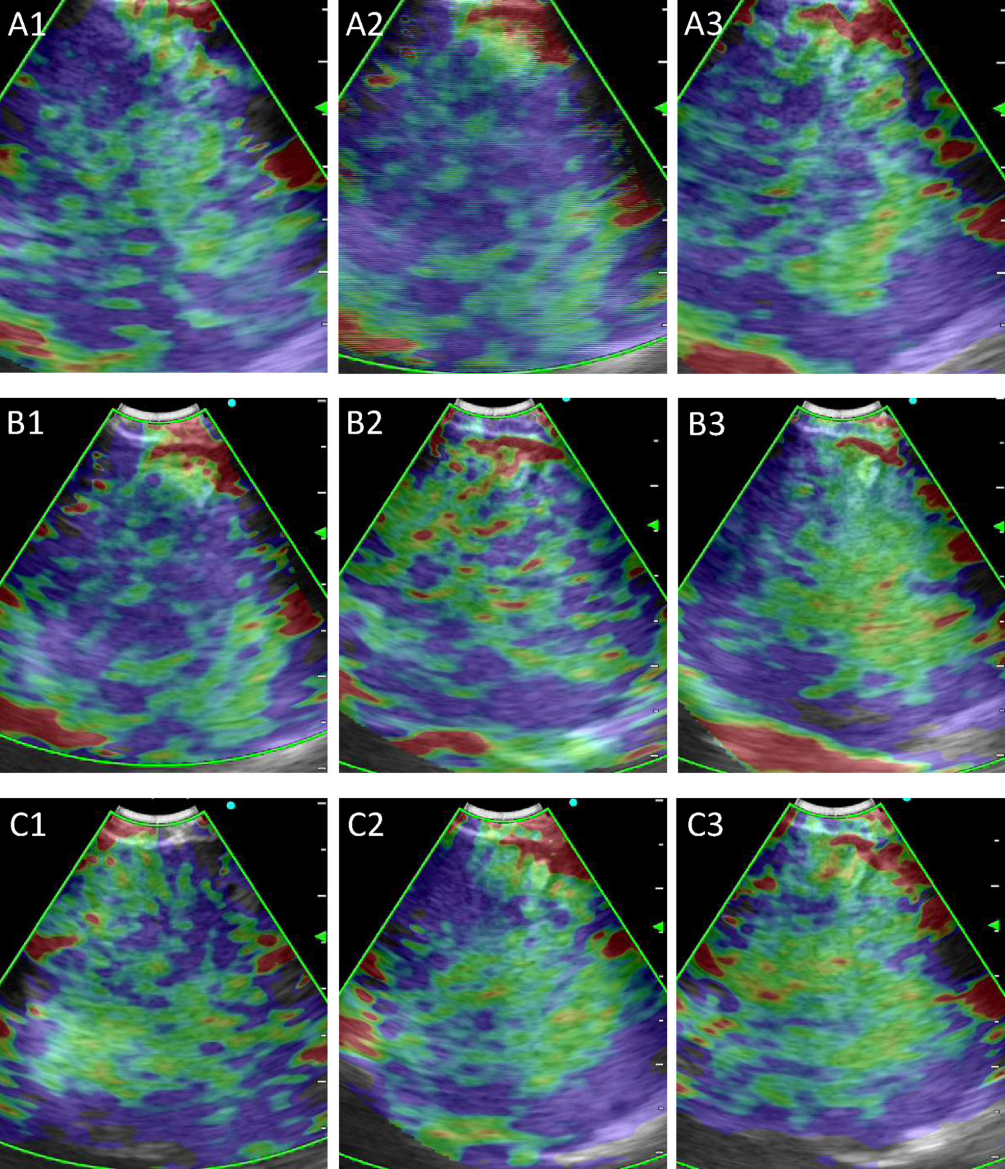
Representative images selected by machine learning, expert and trainee groups. **(A)** 1–3 are representative images selected by the machine learning model; **(B)** 1–3 are representative images selected by the three experts; **(C)** 1–3 are representative images selected by the three trainees.

A total of 415 LNs from 351 patients (247 men, 104 women; age: 60.45 ± 11.31 years) were analyzed in the training and validation sets, and 91 LNs from 73 patients (52 men, 21 women; age:58.82 ± 10.95 years) were used as the test set (**Table 1**). 311 LNs were included in the training set and 104 LNs in the validation set. Malignant LNs accounted for 61.69% in the training and validation sets and 58.24% in the test set. **Figure 3** displayed the representative images selected by machine learning, expert and trainee groups.

**Table 1:** Characteristic of LNs in the training, validation and test sets.

Characteristic of LNs	Training and validation sets No. (%)	Test set No. (%)
**EBUS size**		
Long axis, mean ± SD, mm	21.55 ± 6.71	22.48 ± 7.18
Short axis, mean ± SD, mm	17.90 ± 9.56	17.23 ± 6.45
**CT size^※^**		
Long axis, mean ± SD, mm	25.50 ± 9.48	24.35 ± 8.49
Short axis, mean ± SD, mm	16.70 ± 6.73	16.45 ± 7.02
**Station**		
2L	1 (0.24)	0 (0.00)
2R	8 (1.93)	1 (1.10)
3P	2 (0.48)	0 (0.00)
4L	19 (4.58)	7 (7.69)
4R	135 (32.53)	30 (32.97)
7	160 (38.55)	26 (28.57)
10L	2 (0.48)	1 (1.10)
10R	3 (0.72)	1 (1.10)
11L	32 (7.71)	10 (10.99)
11Rs	32 (7.71)	5 (5.49)
11Ri	19 (4.58)	10 (10.99)
12L	1 (0.24)	0 (0.00)
12R	1 (0.24)	0 (0.00)
**Diagnosis**		
**Malignant**	256 (61.69)	53 (58.24)
Adenocarcinoma	110 (26.51)	25 (27.47)
Squamous carcinoma	39 (9.40)	5 (5.49)
NSCLC-NOS	13 (3.13)	4 (4.40)
Small cell lung cancer	60 (14.46)	15 (16.48)
Large cell neuroendocrine carcinoma	1 (0.24)	0 (0.00)
NET-NOS	11 (2.65)	2 (2.20)
Unknown type of lung cancer	13 (3.13)	1 (1.10)
Carcinosarcoma	1 (0.24)	0 (0.00)
Lymphoma	3 (0.72)	0 (0.00)
Metastatic tumors (non-lung primary malignancy)	5 (1.20)	1 (1.10)
**Benign**	159 (38.31)	38 (41.76)
Nonspecific lymphadenitis	97 (23.37)	16 (17.58)
Sarcoidosis	53 (12.77)	15 (16.48)
Tuberculosis	9 (2.17)	7 (7.69)

^※^The size of LNs on CT images was measured on 393 LNs in the training and validation sets and 88 LNs in the test set. A total of 25 LNs were missing on CT in both groups.

LNs, lymph nodes; NSCLC-NOS, non-small cell lung cancer not otherwise specified; NET-NOS, neuroendocrine tumor not otherwise specified.

### Stability and Diagnostic Performance Analysis by the Qualitative Grading Score

To evaluate the stability of machine learning selected images, we analyzed the differences between the three pictures of machine learning, expert and trainee groups, respectively. Results demonstrated that there was a statistical difference in the expert group, while the images of machine learning and trainee groups were relatively stable (**Table 2**). Besides, diagnostic performance in **Table 3** showed that the diagnostic accuracies of machine learning group were 82.42, 79.12 and 75.82% respectively, slightly lower than experts (p = 0.121), but significantly higher than trainee group (p <0.001).

**Table 2:** Differences between images within each group by qualitative grading score.

	p value		
	Machine learning group	Expert group	Trainee group
Image 123	0.210	0.036	0.205
Image 12	0.134	0.058	0.862
Image 13	0.088	0.029	0.105
Image 23	0.637	0.366	0.059

**Table 3:** Diagnostic efficiency of the three groups by qualitative grading score.

Group	Sen	Spe	PPV	NPV	Acc	FPR	FNR
Machine learning 1	84.91%	78.95%	84.91%	78.95%	82.42%	21.05%	15.09%
Machine learning 2	83.02%	73.68%	81.48%	75.68%	79.12%	26.32%	16.98%
Machine learning 3	79.25%	71.05%	79.25%	71.05%	75.82%	28.95%	20.75%
Expert 1	92.45%	73.68%	83.05%	87.50%	84.62%	26.32%	7.55%
Expert 2	90.57%	73.68%	82.76%	84.85%	83.52%	26.32%	9.43%
Expert 3	88.68%	78.95%	85.45%	83.33%	84.62%	21.05%	11.32%
Trainee 1	62.26%	60.53%	68.75%	53.49%	61.54%	39.47%	37.74%
Trainee 2	64.15%	71.05%	75.56%	58.70%	67.03%	28.95%	35.85%
Trainee 3	69.81%	60.53%	71.15%	58.97%	65.93%	39.47%	30.19%

Sen, sensitivity; Spe, specificity; PPV, positive predictive value; NPV, negative predictive value; Acc, accuracy; FPR, false positive rate; FNR, false negative rate.

### Stability Analysis by Quantitative Methods

In order to assess machine learning selected images more objectively, paired t-test was conducted on quantitative results of machine learning and expert groups. No statistical difference between the two groups was found which demonstrated that images selected by machine learning can reach the expert level (**Table 4**). In terms of the stability analysis of the images within and between the three groups, **Table 5** showed that CV values of machine learning group were lower than expert and trainee groups for each indicator, and among which the trainee group had the highest CV values. Besides, for SAR and B/G, there were statistical differences between machine learning and the other two groups, indicating that machine learning selected images in the test set were more stable than those selected by expert and trainee groups (**Table 5**).

**Table 4:** Comparison of quantitative mean values between machine learning and expert groups.

Variable	p value
SAR	0.801
B/G	0.693
Mean hue value	0.862
Mean gray value	0.514

SAR, stiff area ratio; B/G, elasticity ratio of blue/green.

**Table 5:** Differences in CV values of quantitative methods among the three groups.

Indicator	Machine learning (mean ± SD)	Experts(mean ± SD)	Trainees(mean ± SD)	Machine learning *vs*. Experts(p value)	Machine learning *vs*. Trainees(p value)	Experts *vs*. Trainees(p value)
SAR	0.127 ± 0.109	0.167 ± 0.124	0.200 ± 0.156	2.18E−03	3.22E−06	5.68E−02
B/G	0.079 ± 0.061	0.105 ± 0.067	0.127 ± 0.070	6.69E−03	2.72E−06	2.86E−02
Mean hue value	0.036 ± 0.029	0.042 ± 0.024	0.053 ± 0.036	1.44E−01	7.57E−05	9.50E−03
Mean gray value	0.013 ± 0.044	0.020 ± 0.062	0.022 ± 0.062	3.96E−01	2.56E−01	8.07E−01

CV, coefficient of variation; SD, standard deviation; SAR, stiff area ratio; B/G, elasticity ratio of blue/green.

### Diagnostic Performance Analysis by Quantitative Methods

**Figure 4:**
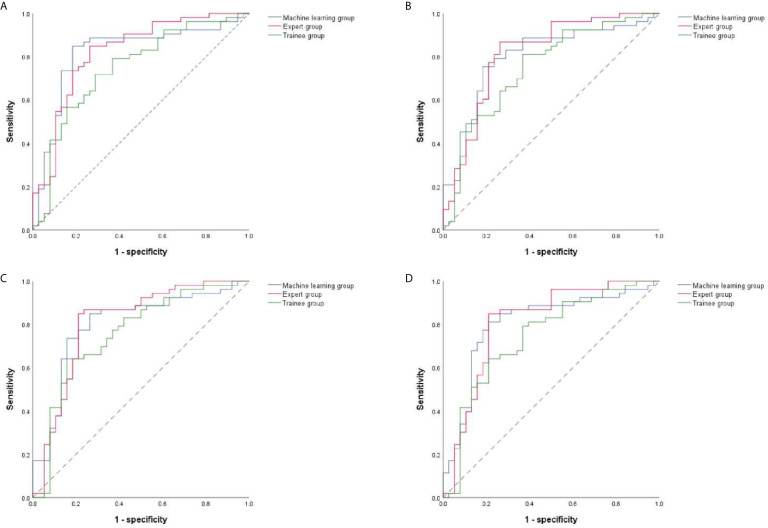
ROC curves of four quantitative methods for machine learning, expert and trainee groups. **(A–D)** illustrate four quantitative indicators including SAR, B/G, mean hue value and mean gray value, respectively. ROC, receiver operating characteristic; SAR, stiff area ratio; B/G, elasticity ratio of blue/green.

The ROC curves were performed on quantitative results of the three groups and cut-off values with the best diagnostic performances were drawn (**Figure 4**). **Table 6** reflected that the accuracies of four quantitative methods including SAR, B/G, mean hue value and mean gray value in the machine learning group were 83.52%, 78.01%, 80.22% and 80.22% respectively. Correspondingly the expert groups were 80.22%, 81.32%, 82.42% and 82.42% respectively. By contrast, the best indicator in the trainee group was B/G, with the highest accuracy of only 73.63%.

**Table 6:** Diagnostic efficiency of the three groups by quantitative methods.

Group	AUC	Cut-off	Sen	Spe	PPV	NPV	Acc
**Machine learning**							
SAR	0.819	0.402	84.91%	81.58%	86.54%	79.49%	83.52%
B/G	0.798	1.176	75.47%	81.58%	85.11%	70.45%	78.02%
Mean hue value	0.801	133.762	84.91%	73.68%	81.82%	77.78%	80.22%
Mean gray value	0.805	194.632	81.13%	78.95%	84.31%	75.00%	80.22%
**Expert**							
SAR	0.822	0.403	84.91%	73.68%	81.82%	77.78%	80.22%
B/G	0.812	1.116	86.79%	73.68%	82.14%	80.00%	81.32%
Mean hue value	0.808	134.870	84.91%	78.95%	84.91%	78.95%	82.42%
Mean gray value	0.809	194.329	84.91%	78.95%	84.91%	78.95%	82.42%
**Trainee**							
SAR	0.746	0.452	71.70%	71.05%	77.55%	64.29%	71.43%
B/G	0.750	1.043	81.13%	63.16%	75.44%	70.59%	73.63%
Mean hue value	0.758	139.811	64.15%	84.21%	85.00%	62.75%	72.53%
Mean gray value	0.744	195.976	64.15%	78.95%	80.95%	61.22%	70.33%

SAR, stiff area ratio; B/G, elasticity ratio of blue/green; AUC, area under curve; Sen, sensitivity; Spe, specificity; PPV, positive predictive value; NPV, negative predictive value; Acc, accuracy.

## Discussion

Lung cancer is the leading cause of cancer associated morbidity and mortality around the world (36). Pulmonary diseases can be diagnosed by draining LNs, therefore the diagnosis of intrathoracic LNs is related to subsequent treatment strategies. EBUS strain elastography imaging is a useful noninvasive tool in differentiating benign from malignant LNs. The machine learning algorithm was used to automatically select representative images from the EBUS strain elastography videos in this study and the image quality was equivalent to the expert level.

Traditional qualitative methods are convenient for clinical application, but subjectivity and the difference in experience between different doctors can affect the accurate diagnosis. Images used for quantitative analysis are still manually selected which cannot avoid subjectivity. The CV values in **Table 5** reflect the instability of manual selection, and the images selected by doctors with different experience had various quality. For qualitative results, there was a statistical difference between the images selected by experts (p = 0.036) but not by trainees (p = 0.205) (**Table 2**). However, a bigger difference presented in diagnostic accuracies among trainees than experts. This was because the diagnosis performance was calculated based on the dichotomy, that is, 1–3 were classified as benign and 4–5 as malignant, yet the differences of qualitative score were counted according to the five categories. Besides, regarding the diagnostic performance among the three groups, the qualitative diagnostic performance of expert group was the highest in the whole study. However, the quantitative results were similar to that of machine learning group, possibly due to the subjectivity of qualitative assessment among different experts. Compared with the qualitative results, the quantitative methods can evaluate the image quality selected by machine learning more objectively. Elastography can only reflect the relative hardness of target lesion, and fibrosis within sarcoidosis may result in stiffer tissue and necrosis within malignant LNs may lead to softer lesions (37, 38). Thus, the highest diagnostic accuracy of automatic image selection model by qualitative and quantitative methods can only reach 83.52%, which was not only due to inaccurate image selection but also the property of the lesion itself. In addition to the four quantitative methods used in this study, strain ratio and strain histogram are also quantitative methods and study found that strain histogram showed better predictive value than strain ratio with a diagnostic rate of 82% in malignant LNs prediction (39). It can be seen that different quantitative methods can lead to various diagnostic results, and there is no unified quantitative method at present. In this study, different results were produced by the four methods in the three groups, but the quality of the images had more effect than the quantitative method on the final results. Notably, the machine learning algorithm in this study was valid for representative images selection of EBUS strain elastography videos, but the implementation of this algorithm needed integration by the manufacturer to become clinically applicable.

This study still had some limitations. Since there was no restriction on the type of disease included, the machine learning model was only suitable for the diagnosis of intrathoracic LNs enlargement, and further studies were need to determine whether or not this technique is valid to the stage of lung cancer. Besides, although high-quality images were selected from elastography videos, no diagnosis was made by the model for these images, and EBUS modes of grayscale and blood flow Doppler were not applied. The automatic EBUS multimodal image selection and diagnosis may be more convenient for clinical application. Moreover, this was a single-center retrospective study with limited number of LNs and some diseases accounted for limited proportions. Prospective studies and more LNs to train, validate and test the model may acquire more stable models and more convincing results. Thus, it was worthwhile to carry out multi-center studies to improve the outcome of the model (40).

In conclusion, through the application of machine learning algorithm to EBUS strain elastography, we realized the automatic selection of high-quality and stable images from strain elastography videos. The automatic image selection model needs further prospective clinical validation and has potential value in guiding the diagnosis of intrathoracic LNs.

## Data Availability Statement

The original contributions presented in the study are included in the article/**Supplementary Material**. Further inquiries can be directed to the corresponding authors.

## Ethics Statement

The studies involving human participants were reviewed and approved by Ethics Committee of Shanghai Chest Hospital. Written informed consent for participation was not required for this study in accordance with the national legislation and the institutional requirements. Written informed consent was not obtained from the individual(s) for the publication of any potentially identifiable images or data included in this article.

## Author Contributions

XZ collected videos, selected representative images, conducted qualitative and quantitative analysis of LNs, and performed statistical analysis. JL designed the machine learning model for automatic selection of representative images. JC and XZ evaluated images selected by machine learning. FX, LW, and JS selected representative images and scored them qualitatively as the expert group. JS and WD designed the study and reviewed the manuscript. JS and HX supported this study. All authors contributed to the article and approved the submitted version.

## Funding

This work was supported by National Natural Science Foundation of China (grant numbers 81870078, 61720106001, 61971285, and 61932022), Shanghai Municipal Health and Medical Talents Training Program (grant number 2018BR09), Shanghai Municipal Education Commission-Gaofeng Clinical Medicine Grant Support (grant number 20181815), and the Program of Shanghai Academic Research Leader (grant 17XD1401900).

## Conflict of Interest

The authors declare that the research was conducted in the absence of any commercial or financial relationships that could be construed as a potential conflict of interest.

The reviewer MC declared a shared affiliation with the authors to the handling editor at time of review.

## References

[1] Silvestri GA Gonzalez AV Jantz MA Margolis ML Gould MK Tanoue LT. Methods for Staging non-Small Cell Lung Cancer: Diagnosis and Management of Lung Cancer, 3rd Ed: American College of Chest Physicians Evidence-Based Clinical Practice Guidelines. Chest (2013) 143(5 Suppl):e211S–e50S. 10.1378/chest.12-2355 23649440

[2] Um SW Kim HK Jung SH Han J Lee KJ Park HY. Endobronchial Ultrasound Versus Mediastinoscopy for Mediastinal Nodal Staging of non-Small-Cell Lung Cancer. J Thorac Oncol (2015) 10(2):331–7. 10.1097/JTO.0000000000000388 25611227

[3] Wahidi MM Herth F Yasufuku K Shepherd RW Yarmus L Chawla M. Technical Aspects of Endobronchial Ultrasound-Guided Transbronchial Needle Aspiration: Chest Guideline and Expert Panel Report. Chest (2016) 149(3):816–35. 10.1378/chest.15-1216 26402427

[4] Sun J Zheng X Mao X Wang L Xiong H Herth FJF. Endobronchial Ultrasound Elastography for Evaluation of Intrathoracic Lymph Nodes: A Pilot Study. Respiration (2017) 93(5):327–38. 10.1159/000464253 28324873

[5] Fujiwara T Nakajima T Inage T Sata Y Sakairi Y Tamura H. The Combination of Endobronchial Elastography and Sonographic Findings During Endobronchial Ultrasound-Guided Transbronchial Needle Aspiration for Predicting Nodal Metastasis. Thorac Cancer (2019) 10(10):2000–5. 10.1111/1759-7714.13186 PMC6775026 31474004

[6] Garra BS Cespedes EI Ophir J Spratt SR Zuurbier RA Magnant CM. Elastography of Breast Lesions: Initial Clinical Results. Radiology (1997) 202(1):79–86. 10.1148/radiology.202.1.8988195 8988195

[7] Lyshchik A Higashi T Asato R Tanaka S Ito J Mai JJ. Thyroid Gland Tumor Diagnosis At US Elastography. Radiology (2005) 237(1):202–11. 10.1148/radiol.2363041248 16118150

[8] Nemakayala D Patel P Rahimi E Fallon MB Thosani N. Use of Quantitative Endoscopic Ultrasound Elastography for Diagnosis of Pancreatic Neuroendocrine Tumors. Endoscopic ultrasound (2016) 5(5):342–5. 10.4103/2303-9027.191680 PMC5070294 27803909

[9] Cochlin DL Ganatra RH Griffiths DF. Elastography in the Detection of Prostatic Cancer. Clin Radiol (2002) 57(11):1014–20. 10.1053/crad.2002.0989 12409113

[10] Sandrin L Fourquet B Hasquenoph JM Yon S Fournier C Mal F. Transient Elastography: A New Noninvasive Method for Assessment of Hepatic Fibrosis. Ultrasound Med Biol (2003) 29(12):1705–13. 10.1016/j.ultrasmedbio.2003.07.001 14698338

[11] Okasha HH Mansour M Attia KA Khattab HM Sakr AY Naguib M. Role of High Resolution Ultrasound/Endosonography and Elastography in Predicting Lymph Node Malignancy. Endoscopic ultrasound (2014) 3(1):58–62. 10.4103/2303-9027.121252 24949412 PMC4063258

[12] Ophir J Céspedes I Ponnekanti H Yazdi Y Li X. Elastography: A Quantitative Method for Imaging the Elasticity of Biological Tissues. Ultrasonic Imaging (1991) 13(2):111–34. 10.1177/016173469101300201 1858217

[13] Zaleska-Dorobisz U Kaczorowski K Pawluś A Puchalska A Inglot M. Ultrasound Elastography - Review of Techniques and its Clinical Applications. Adv Clin Exp Med Off Organ Wroclaw Med Univ (2014) 23(4):645–55. 10.17219/acem/26301 25166452

[14] Dietrich CF Jenssen C Herth FJ. Endobronchial Ultrasound Elastography. Endoscopic ultrasound (2016) 5(4):233–8. 10.4103/2303-9027.187866 PMC4989403 27503154

[15] Ozturk A Grajo JR Dhyani M Anthony BW Samir AE. Principles of Ultrasound Elastography. Abdom Radiol (NY) (2018) 43(4):773–85. 10.1007/s00261-018-1475-6 PMC5973820 29487968

[16] Zhi X Chen J Xie F Sun J Herth FJF. Diagnostic Value of Endobronchial Ultrasound Image Features: A Specialized Review. Endoscopic ultrasound (2020) 10(1):3–18. 10.4103/eus.eus_43_20 PMC7980684 32719201

[17] Nakajima T Shingyouji M Nishimura H Iizasa T Kaji S Yasufuku K. New Endobronchial Ultrasound Imaging for Differentiating Metastatic Site Within a Mediastinal Lymph Node. J Thorac Oncol (2009) 4(10):1289–90. 10.1097/JTO.0b013e3181b05713 20197735

[18] Nakajima T Inage T Sata Y Morimoto J Tagawa T Suzuki H. Elastography for Predicting and Localizing Nodal Metastases During Endobronchial Ultrasound. Respiration (2015) 90(6):499–506. 10.1159/000441798 26571232

[19] Abedini A Razavi F Farahani M Hashemi M Emami H Mohammadi F. The Utility of Elastography During EBUS-TBNA in a Population With a High Prevalence of Anthracosis. Clin Respir J (2020) 14(5):488–94. 10.1111/crj.13159 32034995

[20] Mao XW Yang JY Zheng XX Wang L Zhu L Li Y. [Comparison of Two Quantitative Methods of Endobronchial Ultrasound Real-Time Elastography for Evaluating Intrathoracic Lymph Nodes]. Zhonghua Jie He He Hu Xi Za Zhi (2017) 40(6):431–4. 10.3760/cma.j.issn.1001-0939.2017.06.007 28592025

[21] Săftoiu A Vilmann P Hassan H Gorunescu F. Analysis of Endoscopic Ultrasound Elastography Used for Characterisation and Differentiation of Benign and Malignant Lymph Nodes. Ultraschall Med (2006) 27(6):535–42. 10.1055/s-2006-927117 17160759

[22] Săftoiu A Vilmann P Ciurea T Popescu GL Iordache A Hassan H. Dynamic Analysis of EUS Used for the Differentiation of Benign and Malignant Lymph Nodes. Gastrointest Endosc (2007) 66(2):291–300. 10.1016/j.gie.2006.12.039 17643702

[23] Landoni V Francione V Marzi S Pasciuti K Ferrante F Saracca E. Quantitative Analysis of Elastography Images in the Detection of Breast Cancer. Eur J Radiol (2012) 81(7):1527–31. 10.1016/j.ejrad.2011.04.012 21530123

[24] da Silva GLF Valente TLA Silva AC de Paiva AC Gattass M. Convolutional Neural Network-Based PSO for Lung Nodule False Positive Reduction on CT Images. Comput Methods Programs BioMed (2018) 162:109–18. 10.1016/j.cmpb.2018.05.006 29903476

[25] Esteva A Kuprel B Novoa RA Ko J Swetter SM Blau HM. Dermatologist-Level Classification of Skin Cancer With Deep Neural Networks. Nature (2017) 542(7639):115–8. 10.1038/nature21056 PMC8382232 28117445

[26] Gulshan V Peng L Coram M Stumpe MC Wu D Narayanaswamy A. Development and Validation of a Deep Learning Algorithm for Detection of Diabetic Retinopathy in Retinal Fundus Photographs. JAMA (2016) 316(22):2402–10. 10.1001/jama.2016.17216 27898976

[27] Min JK Kwak MS Cha JM. Overview of Deep Learning in Gastrointestinal Endoscopy. Gut Liver (2019) 13(4):388–93. 10.5009/gnl18384 PMC6622562 30630221

[28] Misawa M Kudo SE Mori Y Takeda K Maeda Y Kataoka S. Accuracy of Computer-Aided Diagnosis Based on Narrow-Band Imaging Endocytoscopy for Diagnosing Colorectal Lesions: Comparison With Experts. Int J Comput Assist Radiol Surg (2017) 12(5):757–66. 10.1007/s11548-017-1542-4 28247214

[29] Benz M Rojas-Solano JR Kage A Wittenberg T Munzenmayer C Becker HD. Computer-Assisted Diagnosis for White Light Bronchoscopy: First Results. Chest (2010) 138(4):433A. 10.1378/chest.10959

[30] Feng PH Lin YT Lo CM. A Machine Learning Texture Model for Classifying Lung Cancer Subtypes Using Preliminary Bronchoscopic Findings. Med Phys (2018) 45(12):5509–14. 10.1002/mp.13241 30325517

[31] Novak C Shafer SJPICSCoCV Recognition P. Anatomy of a Color Histogram. Proc 1992 IEEE Comput Soc Conf Comput Vision Pattern Recognit (1992) 599–605. 10.1109/CVPR.1992.223129

[32] Arandjelovi R Gronát P Torii A Pajdla T Sivic JJIToPA Intelligence M. Netvlad: CNN Architecture for Weakly Supervised Place Recognition. IEEE Trans Pattern Anal Mach Intell (2018) 40:1437–51. 10.1109/TPAMI.2017.2711011 28622667

[33] Jégou H Douze M Schmid C PJICSCoCV Pérez Recognition P. Aggregating Local Descriptors Into a Compact Image Representation. IEEE Comput Soc Conf Comput Vision Pattern Recognit (2010) 3304–11. 10.1109/CVPR.2010.5540039

[34] Lin R Xiao J Fan JJA. Nextvlad: An Efficient Neural Network to Aggregate Frame-Level Features for Large-scale Video Classification. ArXiv (2018). 10.1007/978-3-030-11018-5_19

[35] Yeung M Yeo B Liu BJCVIU. Segmentation of Video by Clustering and Graph Analysis. Comput Vis Image Underst (1998) 71:94–109. 10.1006/cviu.1997.0628

[36] Bray F Ferlay J Soerjomataram I Siegel RL Torre LA Jemal A. Global Cancer Statistics 2018: GLOBOCAN Estimates of Incidence and Mortality Worldwide for 36 Cancers in 185 Countries. CA: Cancer J Clin (2018) 68(6):394–424. 10.3322/caac.21492 30207593

[37] Livi V Cancellieri A Pirina P Fois A van der Heijden E Trisolini R. Endobronchial Ultrasound Elastography Helps Identify Fibrotic Lymph Nodes in Sarcoidosis. Am J Respir Crit Care Med (2019) 199(3):e24–e5. 10.1164/rccm.201710-2004IM 30207738

[38] Lin CK Yu KL Chang LY Fan HJ Wen YF Ho CC. Differentiating Malignant and Benign Lymph Nodes Using Endobronchial Ultrasound Elastography. J Formos Med Assoc (2019) 118(1 Pt 3):436–43. 10.1016/j.jfma.2018.06.021 30007831

[39] Verhoeven RLJ de Korte CL van der Heijden E. Optimal Endobronchial Ultrasound Strain Elastography Assessment Strategy: An Explorative Study. Respiration (2019) 97(4):337–47. 10.1159/000494143 PMC6492606 30554224

[40] Verhoeven RLJ Trisolini R Leoncini F Candoli P Bezzi M Messi A. Predictive Value of Endobronchial Ultrasound Strain Elastography in Mediastinal Lymph Node Staging: The E-Predict Multicenter Study Results. Respiration (2020) 99:484–92. 10.1159/000507592 32492682

