# Supplementary figures and images for: Automatic Image Selection Model Based on Machine Learning for Endobronchial Ultrasound Strain Elastography Videos
Source: Front Oncol. 2021 May 31;11:673775. doi: 10.3389/fonc.2021.673775 (PMC8201408; doi:10.3389/fonc.2021.673775)

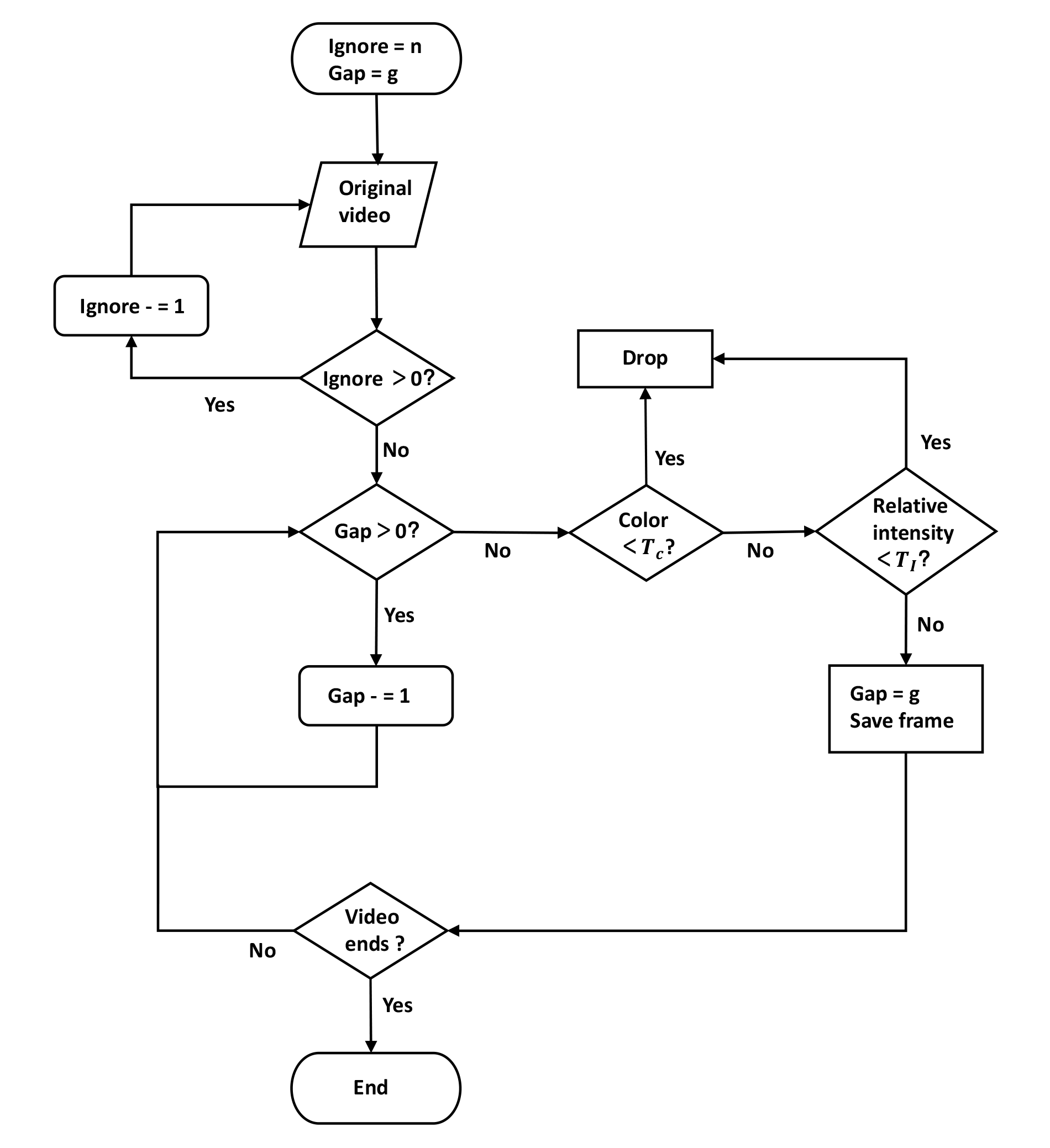

Supplement: Supplementary file 1 [file Image_1.tif]
