# Supplementary material for: Automatic Image Selection Model Based on Machine Learning for Endobronchial Ultrasound Strain Elastography Videos
Source: Front Oncol. 2021 May 31;11:673775. doi: 10.3389/fonc.2021.673775 (PMC8201408; doi:10.3389/fonc.2021.673775)
Supplement: Supplementary file 2 [file DataSheet_1.docx]

Supplementary Material

# Supplementary Methods: The low-complexity empirical algorithm designed for the video to eliminate redundant frames

To drop uncolored frames that the hardness of the targeted lymph node (LN) was not illustrated successfully and where images that only a small part of the LN was imaged, we propose an indicator named colored ratio, and symbolize it as $r$. Mathematically, it is defined as:

$$r=\frac{\sum_{i,j}^{M,N} I_{ij}^{E}}{MN}, I_{ij}^{E}=\left\{ \begin{aligned} 1 if S_{ij}\geq T_{s} \\ 0 if S_{ij}<T_{s} \end{aligned} \right.$$

Where $M,N$ is the width and height of the given frame, $S$ is the saturation of the given frame, and $T$ is the empirical threshold (we apply $T=0.75$ here). $I_{ij}^{E}$ is the indicator variable for event $S_{ij}\geq T$. With indicator $r$, valid colored frames and other frames can be separated according to an empirical threshold.

Development of elastic image is a process from dark to bright gradually. For frames at the start stage, images may be covered by a large proportion of colored pixels but with low mean intensity. Besides, different elastic videos have different contrasts, which means a fixed threshold for intensity cannot fit all videos well. To adapt to the varying contrast of different videos, an adaptive intensity rather than a fixed intensity should be used to measure the quality of frames. To drop valid colored frames presenting low illuminance and involves artifacts. We propose another indicator named relative intensity to separate valid colored frames further and symbolize it as $v$. Mathematically, it is defined as:

$$v=R*\frac{\sum_{i,j}^{M,N} I_{ij}^{E}V_{ij}}{\sum_{i,j}^{M,N} I_{ij}^{E}}*\frac{\sum_{i,j}^{M,N} 1-I_{ij}^{E}}{\sum_{i,j}^{M,N} \left( 1-I_{ij}^{E} \right)V_{ij}}$$

Where $R$ (we apply $R=90$ here) is the reference intensity, and $V$ is the value (i.e. the value channel in HSV view) of the given frame. In summary, given a frame $a$, if $r_{a}>T_{r}$ and $v_{a}>T_{v}$, $a$ will be regarded as qualified.

# Supplementary Table 1. Qualitative evaluation results of hyperparameters on the validation sets.

| Hyper-parameters | | Sensitivity | Specificity | PPV | NPV | Accuracy |
| --- | --- | --- | --- | --- | --- | --- |
| Run twice | Num cluster |  |  |  |  |  |
| FALSE | 32 |  |  |  |  |  |
| Figure 1 | | 78.13% | 60.00% | 75.76% | 63.16% | 71.15% |
| Figure 2 | | 73.44% | 70.00% | 79.66% | 62.22% | 72.12% |
| Figure 3 | | 78.13% | 67.50% | 79.37% | 65.85% | 74.04% |
| Mean value | | 76.56% | 65.83% | 78.26% | 63.74% | 72.44% |
| FALSE | 64 |  |  |  |  |  |
| Figure 1 | | 85.94% | 57.50% | 76.39% | 71.88% | 75.00% |
| Figure 2 | | 76.56% | 62.50% | 76.56% | 62.50% | 71.15% |
| Figure 3 | | 85.94% | 55.00% | 75.34% | 70.97% | 74.04% |
| Mean value | | 82.81% | 58.33% | 76.10% | 68.45% | 73.40% |
| FALSE | 128 |  |  |  |  |  |
| Figure 1 | | 76.56% | 52.50% | 72.06% | 58.33% | 67.31% |
| Figure 2 | | 75.00% | 55.00% | 72.73% | 57.89% | 67.31% |
| Figure 3 | | 81.25% | 55.00% | 74.29% | 64.71% | 71.15% |
| Mean value | | 77.60% | 54.17% | 73.02% | 60.31% | 68.59% |
| TRUE | 32 |  |  |  |  |  |
| Figure 1 | | 79.69% | 57.50% | 75.00% | 63.89% | 71.15% |
| Figure 2 | | 79.69% | 62.50% | 77.27% | 65.79% | 73.08% |
| Figure 3 | | 76.56% | 70.00% | 80.33% | 65.12% | 74.04% |
| Mean value | | 78.65% | 63.33% | 77.53% | 64.93% | 72.76% |
| TRUE | 64 |  |  |  |  |  |
| Figure 1 | | 78.13% | 62.50% | 76.92% | 64.10% | 72.12% |
| Figure 2 | | 79.69% | 67.50% | 79.69% | 67.50% | 75.00% |
| Figure 3 | | 81.25% | 67.50% | 80.00% | 69.23% | 75.96% |
| Mean value | | 79.69% | 65.83% | 78.87% | 66.94% | 74.36% |
| TRUE | 128 |  |  |  |  |  |
| Figure 1 | | 79.69% | 57.50% | 75.00% | 63.89% | 71.15% |
| Figure 2 | | 78.13% | 52.50% | 72.46% | 60.00% | 68.27% |
| Figure 3 | | 81.25% | 55.00% | 74.29% | 64.71% | 71.15% |
| Mean value | | 79.69% | 55.00% | 73.92% | 62.86% | 70.19% |

PPV, positive predictive value; NPV, negative predictive value

# Supplementary Table 2. Quantitative evaluation results of hyperparameters on the validation sets.

| Hyper-parameters | | Sensitivity | Specificity | PPV | NPV | Accuracy |
| --- | --- | --- | --- | --- | --- | --- |
| Run twice | Num cluster |  |  |  |  |  |
| FALSE | 32 |  |  |  |  |  |
| SAR | | 84.38% | 62.50% | 78.26% | 71.43% | 75.96% |
| B/G | | 84.38% | 62.50% | 78.26% | 71.43% | 75.96% |
| Mean hue value | | 68.75% | 82.50% | 86.27% | 62.26% | 74.04% |
| Mean gray value | | 73.44% | 75.00% | 82.46% | 63.83% | 74.04% |
| FALSE | 64 |  |  |  |  |  |
| SAR | | 59.38% | 85.00% | 86.36% | 56.67% | 69.23% |
| B/G | | 84.38% | 60.00% | 77.14% | 70.59% | 75.00% |
| Mean hue value | | 75.00% | 72.50% | 81.36% | 64.44% | 74.04% |
| Mean gray value | | 90.63% | 52.50% | 75.32% | 77.78% | 75.96% |
| FALSE | 128 |  |  |  |  |  |
| SAR | | 57.81% | 82.50% | 84.09% | 55.00% | 67.31% |
| B/G | | 46.88% | 90.00% | 88.24% | 51.43% | 63.46% |
| Mean hue value | | 45.31% | 95.00% | 93.55% | 52.05% | 64.42% |
| Mean gray value | | 48.44% | 92.50% | 91.18% | 52.86% | 65.38% |
| TRUE | 32 |  |  |  |  |  |
| SAR | | 79.69% | 62.50% | 77.27% | 65.79% | 73.08% |
| B/G | | 87.50% | 52.50% | 74.67% | 72.41% | 74.04% |
| Mean hue value | | 81.25% | 60.00% | 76.47% | 66.67% | 73.08% |
| Mean gray value | | 87.50% | 52.50% | 74.67% | 72.41% | 74.04% |
| TRUE | 64 |  |  |  |  |  |
| SAR | | 73.44% | 80.00% | 85.45% | 65.31% | 75.96% |
| B/G | | 76.56% | 77.50% | 84.48% | 67.39% | 76.92% |
| Mean hue value | | 73.44% | 80.00% | 85.45% | 65.31% | 75.96% |
| Mean gray value | | 75.00% | 80.00% | 85.71% | 66.67% | 76.92% |
| TRUE | 128 |  |  |  |  |  |
| SAR | | 68.75% | 72.50% | 80.00% | 59.18% | 70.19% |
| B/G | | 79.69% | 60.00% | 76.12% | 64.86% | 72.12% |
| Mean hue value | | 70.31% | 70.00% | 78.95% | 59.57% | 70.19% |
| Mean gray value | | 68.75% | 72.50% | 80.00% | 59.18% | 70.19% |

SAR, stiff area ratio; B/G, elasticity ratio of blue/green; PPV, positive predictive value; NPV, negative predictive value

# Supplementary Figure 1. The algorithm used to extract eligible frames from videos.

Parameters “*Ignore*=n” and “*Gap*=g” are set initially. Then, we make “*Ignore*” minus 1 for each frame until “*Ignore*” equal to 0. This aims at dropping the first n frames in videos. Then, we do the same thing to *Gap* but reset it to g once *Gap* equal to 0. This operation aims at sampling every g frames from videos. For sampled frames, we keep those whose ratio of the colored area exceeds $T_{c}$ and relative intensity exceeds $T_{I}$. “n=1”, “g=1”, “$T_{c}$=0.75”, and “$T_{I}$=65” are applied here.
